# Quality Improvement Project for the Management of per Vaginal (PV) Bleeding in Early Pregnancy: Evaluating the Effectiveness of Patient Information Leaflets

**DOI:** 10.7759/cureus.98922

**Published:** 2025-12-10

**Authors:** Sajad Hussain, Karthik Kittappa

**Affiliations:** 1 Ophthalmology, Mid Yorkshire Teaching Trust, Wakefield, GBR; 2 Emergency Medicine, Mid Yorkshire Teaching Trust, Wakefield, GBR

**Keywords:** clinical audit system, emergency department attendances, leaflet, quality improvment project, vaginal bleed

## Abstract

Vaginal bleeding in early pregnancy is a frequent cause of emergency department (ED) visits, often associated with significant anxiety. Effective communication and the provision of written information are considered fundamental to patient-centred care and are consistently emphasised across clinical guidelines and best-practice recommendations. This study aimed to assess compliance with national and local standards for communication and information provision to women presenting with per vaginal (PV) bleeding in early pregnancy. A two-cycle retrospective audit was conducted in the ED of a district general hospital in Yorkshire, England. Case notes of women presenting with PV bleeding (≤16 weeks’ gestation) between March and June 2025 were reviewed. Standards assessed included provision of patient information leaflets (PILs), verbal explanations, and confirmation of understanding. Following the first audit, interventions included staff education, improved leaflet accessibility, and visual reminders. A re-audit was conducted three months later using identical criteria. A total of 217 patients were reviewed (119 in the first audit, 98 in the second). Leaflet provision improved from 19% to 81% (p < 0.001), verbal explanation from 25% to 75% (p < 0.001), and confirmation of understanding from 25% to 80% (p < 0.001). All showed strong effect sizes (Cramer’s V 0.51-0.60).

Targeted education and improved leaflet access significantly enhanced compliance with communication standards. Simple, low-cost interventions can substantially improve patient understanding, satisfaction, and adherence to national guidelines in the management of early pregnancy bleeding.

## Introduction

Vaginal bleeding in early pregnancy is a common reason for visits to the emergency department (ED), affecting about one in five pregnancies during the first trimester [1]. This often leads to significant anxiety and distress for patients and their families. The initial interaction and communication quality during these encounters are crucial for delivering compassionate and effective care.

Effective communication and accurate information are foundational to good patient-centred care, especially in sensitive, emotionally charged situations like per vaginal (PV) bleeding. In the ED, patients frequently face increased environmental anxiety on top of their medical distress, which can hinder their understanding and memory of verbal information [2]. Multiple studies have shown that patients often recall less than half of what is explained verbally during stressful medical encounters [2,3]. Providing written information, such as patient information leaflets (PILs), can be a vital supplement to verbal explanations. PILs allow patients to review information at their own pace, discuss it with family members for advice, and reinforce their understanding of their diagnosis, management, and follow-up plans. Multiple studies have shown that PILs improve patient knowledge, satisfaction, engagement, and follow-up compliance, especially in distressing situations [4-6]. According to the National Institute for Health and Care Excellence (NICE) guideline NG 126: Ectopic Pregnancy and Miscarriage (2019), all women with early pregnancy complications should receive both verbal and written information [7]. However, adherence to these guidelines remains inconsistent. Barriers include time constraints, unavailability of leaflets, and staff unawareness of the importance of written communication [8]. Addressing these issues presents a simple but effective opportunity to enhance patient experience and safety.

The audit reviewed how early pregnancy PV bleeding was managed at a district hospital, focusing on the information provided to patients and the correct referral to the Early Pregnancy Assessment Unit (EPAU). After introducing staff training and routine PILs, a second audit assessed the improvements.

## Materials and methods

Study design and setting

This was a two-cycle retrospective clinical audit conducted in the EPAU at Mid Yorkshire Teaching Trust, a district general hospital in Yorkshire, England. The EPAU provides assessment and management for women presenting with symptoms such as vaginal bleeding and abdominal pain in early pregnancy (≤16 weeks of gestation).

The audit was registered with the hospital’s clinical audit department and classified as a quality-improvement activity; therefore, formal research ethics approval was not required.

The Plan-Do-Study-Act (PDSA) cycle, which was followed, is summarised in Table 1.

**Table 1:** Plan–Do–Study–Act (PDSA) cycle NICE: National Institute for Health and Care Excellence

Stage	Description
Plan	Audit identified poor compliance with NICE NG126 standards for communication and provision of patient information to women with early pregnancy bleeding. Only 19% received written information, and verbal explanation was inconsistently documented. Aim: to improve leaflet provision and communication standards to >80%.
Do	Implemented interventions: staff education sessions improved leaflet access in all clinical areas, visual reminders, departmental communication via emails and meetings, and reinforced the importance of documentation and verbal explanation.
Study	Re-audit was conducted three months later using identical standards. Documentation showed significant improvements: leaflet provision 19% → 81%, verbal explanation 25% → 75%, understanding confirmed 25% → 80%. Patient follow-up confirmed improved recall of information and satisfaction.
Act	Actions for next cycle: Maintain leaflet stock, embed standards in induction and mandatory training, and introduce electronic prompts to record leaflet provision. Plan a follow-up audit within 6–12 months to assess sustainability. Future work to include patient satisfaction scoring and multi-site comparison.

Audit standards

The audit criteria were derived from local early pregnancy bleeding policy and relevant national guidelines, including NICE Clinical Guideline 126 (Ectopic Pregnancy and Miscarriage) [8]. The following standards were assessed: 1. 100% of patients presenting with PV bleeding in early pregnancy should be offered a PV bleed information leaflet; 2. 100% of cases should include documented evidence that the leaflet was given and explained; 3. All patients should be advised of red-flag symptoms and follow-up instructions consistent with the leaflet content.

Data collection

A retrospective review of electronic records and clinical notes was undertaken for all consecutive patients who attended the EPAU with PV bleeding between March 2025 and June 2025. Each patient was subsequently contacted by phone. Data collected included demographic details such as age and gestation at presentation, information regarding the patient’s first ED visit for PV bleeding, and the final diagnosis, which could include threatened miscarriage, early pregnancy loss, ectopic pregnancy, or other outcomes. Additionally, documentation was reviewed to determine whether a PIL was provided and whether there was evidence that the leaflet had been discussed or explained to the patient. Data were entered into Microsoft Excel (Microsoft Corporation, Redmond, WA) and analysed. Results were expressed as percentages of total eligible cases.

Intervention phase

Following the baseline audit, the results were discussed with the ED multidisciplinary team. Several improvement strategies were implemented to address the findings. These included staff education sessions emphasising the audit results and the importance of providing PILs; improved access to leaflets through restocking at every clinical bay and the addition of clear signage; placement of visual reminders in clinical rooms; and the use of regular emails, messaging prompts, and verbal reminders to encourage staff to distribute the leaflets. After implementing these measures, a re-audit was conducted three months later using the same standards and methodology.

Data analysis

Pre- and post-intervention compliance rates were compared descriptively. Absolute percentage improvements were calculated. The chi-square test of independence (χ²) was used to determine whether there was a significant association between two categorical variables. Although the chi-square test was used to detect a significant difference, it could not quantify how large or meaningful that difference was. A Cramer’s V value was calculated as an effect size measure to quantify the strength of association between two categorical variables.

## Results

A total of 149 patient records were reviewed in Cycle 1 and 127 in Cycle 2. Telephone follow-up was completed for 119 patients (79.9%) in Cycle 1 and 98 patients (77.2%) in Cycle 2.

Telephone follow-up validation

Telephone validation confirmed low communication adherence in Cycle 1, with only 19% of patients recalling receipt of a PIL and 29.4% recalling a verbal explanation. In Cycle 2, recall increased markedly to 76.5% and 82.0%, respectively (Table 2).

**Table 2:** Patient follow-up outcomes and recall of communication measures.

Variable	Total Contacted	Successfully Contacted (%)	Number of patients successfully contacted
Cycle 1	N = 149	79.9	N = 119
Cycle 2	N = 127	77.2	N = 98

Subgroup analyses

By Clinical Area

Cycle 1 exhibited consistently low documentation performance in all areas (leaflet: 18%-20%; explanation: 23%-28%; understanding: 24%-27%). After the intervention, Cycle 2 showed significant improvement across all domains, with documentation rates surpassing 72% for all measures (Table 3).

**Table 3:** A comparison of results between the emergency department, nurse triage, and consultant triage areas across both audit cycles.

Department Area	Leaflet Given (%)	Explanation (%)	Understanding Confirmed (%)
Cycle 1: Emergency department	18	23	24
Cycle 1: Nurse triage	20	28	27
Cycle 1: Consultant red chair triage	18	25	24
Cycle 2: Emergency department	78	72	77
Cycle 2: Nurse triage	85	79	84
Cycle 2: Consultant red chair triage	79	75	79

Similarly, Cycle 1 showed low communication adherence across all staff roles, while Cycle 2 demonstrated consistent and notable improvement, with compliance rising to 71%-83% across all measures (Table 4).

**Table 4:** Differences by staff role before and after the intervention

Staff Role	Leaflet Given (%)	Explanation (%)	Understanding Confirmed (%)
Cycle 1: Junior Doctor	17	22	23
Cycle 1: Nurse	20	26	25
Cycle 1: Consultant	22	29	28
Cycle 2: Junior Doctor	79	71	76
Cycle 2: Nurse	81	78	82
Cycle 2: Consultant	81	77	83

Significant improvements were noted in all communication metrics (Table 5). Provision of PILs rose from 19.0% to 81.0%, verbal explanations from 25.0% to 75.0%, and confirmation of patient understanding from 25.0% to 80.0%. All changes were statistically significant with large effect sizes (Cramer’s V = 0.51-0.60). 

**Table 5:** Key outcomes across both audit cycles ED: emergency department; PV: per vaginal; EPAU: Early Pregnancy Assessment Unit

Measure	First Audit Yes (%)	95% Confidence Intervals (%)	Second Audit Yes (%)	95% Confidence Intervals (%)	χ²	p-value	Cramer's V
First ED visit for PV bleeding	85.0%	76.7–90.7	87.0%	79.0–92.2	0.04	0.838	0.337819
Leaflet given	19.0%	12.5–27.8	81.0%	72.2–87.5	74.42	<0.05	0.60
Patient verbal explanation provided	25.0%	17.5–34.3	75.0%	65.7–82.5	48.02	<0.05	0.51
Patient understanding confirmed	25.0%	17.5–34.3	80.0%	71.1–86.7	58.47	<0.05	0.54
Referred to EPAU	88.0%	80.2–93.0	90.0%	82.6–94.5	0.05	0.821	0.001753

## Discussion

The re-audit revealed substantial improvements in patient communication and the use of written information. Following staff education sessions and the consistent availability of PILs, all communication-related indicators showed statistically significant improvements. The most notable change was the routine distribution of leaflets, which increased compliance from 19% to 81%. This aligns with NICE NG 126 recommendations [7] and indicates greater awareness and accessibility of patient education materials. Studies confirm that leaflets can enhance understanding and recall, especially during emotionally stressful consultations such as PV bleeding [2, 4]. Subgroup analyses showed that improvements were consistent across departments, staff roles, and over time, indicating widespread and sustainable change. 

This re-audit showed that increased leaflet use likely improved understanding, as they enable patients to review information outside stressful environments, such as an ED. Several studies support this, including Stapleton et al. [2], who found that maternity care leaflets developed with evidence-based content and clear language boost patient confidence and engagement [4]. Garrud et al. [3] also showed that written risk information improves comprehension and satisfaction in gynecological and obstetric care. Medina-Córdoba et al. [4] emphasised that well-structured written information enhances understanding, supports patient autonomy, and facilitates shared decision-making.

However, these audit results could be over- or underestimated because, when patients are asked to recall events, their memories may be inaccurate or influenced by emotion or the passage of time. Telephone follow-up depended on the patient's memory of receiving a leaflet or explanation. Patients in the second cycle might recall the interaction more clearly due to shorter follow-up intervals or better experience, while earlier patients may have forgotten. Emotional distress during early pregnancy bleeding can also impact memory.

Performance bias might have influenced the results because staff could change their behaviour when they know they are being observed or audited. After the first cycle, staff realised that communication practices were under surveillance. This likely temporarily improved behaviour, regardless of the educational intervention. Compliance could decline over time once monitoring ceases. To address this, further audits would be conducted to ensure standards are sustained. 

Improvements in verbal explanations and confirming understanding reflect the positive effects of staff education and awareness efforts. Lippke et al. [5] noted that structured communication strategies in obstetrics significantly enhance patient safety and satisfaction. When verbal explanations are combined with written materials, patient recall, compliance, and satisfaction tend to increase together [8]. The rise in confirming patient understanding from 25% to 80% is a significant step toward safer care and shared decision-making. Written info allows patients to revisit key points at their convenience, improving information retention. These results are consistent with Smith and Cross [6], who reported better knowledge retention and satisfaction when written materials accompany clinician explanations. To sustain these improvements, the department should ensure continuous availability of leaflets, train all staff in their use, and incorporate leaflet provision into routine consultations. Regular re-audits and patient feedback can help maintain quality and compliance. This audit demonstrates that simple, low-cost steps, such as consistently providing PILs and emphasising verbal communication, can significantly enhance the patient experience and adherence to national guidelines. These findings underscore the importance of structured communication and written information in improving care quality for women presenting with PV bleeding in early pregnancy.

Recommendations from the audit

Sustain Leaflet Provision

Ensure printed and electronic versions of patient information leaflets are consistently available in all relevant clinical areas (ED, majors, minors, EPAU, and outpatient settings). Regularly review and update content to reflect current NICE and local trust guidance. Embed communication standards in staff training, departmental induction, and mandatory training sessions to ensure consistency by including guidance on providing verbal explanations, using PILs, and confirming understanding. Promote a multidisciplinary approach, involving nursing and medical staff, to maintain uniform communication standards.

Document Communication Practices

Routinely record leaflet provision and confirmation of understanding in patient notes as part of standard documentation templates. Obtain regular patient feedback: Implement short feedback forms or QR code surveys to assess patient perceptions of clarity, reassurance, and usefulness of the information provided, guiding the resource's effectiveness and ensuring leaflets offer maximum benefit.

Plan for Re-audit

Reassess compliance within six to 12 months to ensure ongoing improvement and identify further opportunities for refinement. Consider expanding the audit scope to include patient-reported outcomes such as satisfaction and anxiety reduction scores

Limitations

This audit has several limitations. First, being a retrospective review, it depends on the quality of documentation, which may not capture all clinical interactions or communications. Some staff members may have provided verbal explanations or distributed leaflets without documenting them. This may result in underestimating compliance. Additionally, since the audit was conducted at a single district general hospital, its findings may not be fully applicable to other settings with different patient groups or resources. Third, the outcome measures focused on processes rather than direct patient feedback, satisfaction, or anxiety reduction, which could have offered a more complete picture of the impact. Fourth, the relatively short three-month gap between audit cycles may not reflect the long-term sustainability of improvements. Lastly, because the intervention included multiple strategies (education, reminders, and improved access), the specific contribution of each cannot be determined. Future audits should include patient-reported outcomes, more extended follow-up periods, and a multi-centred approach to enhance external validity and evaluate ongoing quality improvements.

## Conclusions

This clinical audit showed a significant and statistically proven improvement in how women with early pregnancy-related PV bleeding are communicated with and educated. After introducing targeted actions, such as a leaflet and staff training, compliance with essential quality standards rose notably, especially in leaflet distribution, verbal explanations, and confirming patient understanding. These results support adherence to national clinical guidelines and best-practice recommendations and align with best practices for effective, patient-centred communication. The findings highlight the need to blend verbal and written information in routine care to improve understanding, reduce anxiety, and strengthen doctor-patient interactions. This audit underscores the importance of good communication for safe, compassionate, and high-quality maternity and early pregnancy care. Offering written materials along with clear verbal explanations helps ensure patients are informed and supported, especially during emotional times. The successful outcomes of this re-audit show that small, affordable, sustainable measures can lead to significant, measurable improvements in patient experience and care standards. This improves overall satisfaction with the service, preventing readmissions and saving time, money, and resources by re-examining patients for the same initial complaint. 
